# An Underwater Time Reversal Communication Method Using Symbol-Based Doppler Compensation with a Single Sound Pressure Sensor

**DOI:** 10.3390/s18103279

**Published:** 2018-09-29

**Authors:** Anbang Zhao, Caigao Zeng, Juan Hui, Lin Ma, Xuejie Bi

**Affiliations:** 1 Acoustic Science and Technology Laboratory, Harbin Engineering University, Harbin 150001, China; zhaoanbang@hrbeu.edu.cn (A.Z.); cgzeng@hrbeu.edu.cn (C.Z.); malin@hrbeu.edu.cn (L.M.); bixuejie@hrbeu.edu.cn (X.B.); 2 Key Laboratory of Marine Information Acquisition and Security, Harbin Engineering University, Ministry of Industry and Information Technology, Harbin 150001, China; 3 College of Underwater Acoustic Engineering, Harbin Engineering University, Harbin 150001, China; 4 National Key Laboratory of Science and Technology on Underwater Acoustic Antagonizing, China State Shipbuilding Corporation Systems Engineering Research Institute, Beijing 100036, China

**Keywords:** UWA communication, time reversal mirror, symbol-based Doppler compensation, single pressure sensor

## Abstract

Due to the significant multipath and Doppler effects in the underwater acoustic (UWA) channel, the quality of the received signal is degraded, which seriously affects the performance of UWA communication. The paper proposes a time reversal UWA communication method combined with a symbol-based Doppler compensation (SBDC) technique to solve those problems. A single element time reversal mirror (TRM) is used to realize channel equalization and mitigate the inter-symbol interference (ISI) resulting from multipath propagation. The SBDC technique is subsequently used to compensate Doppler effects in the received signal, thereby reducing the bit error rate (BER) and improving the communication performance. In order to verify the performance of the proposed communication method, some simulations with real sounding channels were performed. Moreover, a field UWA communication experiment was conducted in the Songhua River (Harbin, China). The UWA communication experiment achieves nearly error-free performance with a communication rate of 100 bit/s in the bandwidth of 2 kHz. The results of the experiment demonstrate the feasibility and robustness of the proposed UWA communication method.

## 1. Introduction

Multipath spreads and Doppler effects make UWA channels some of the most challenging wireless communication environments [1,2]. The large multipath spreads cause severe ISI, degrading the quality of communication signal. In addition, the slow speed of sound propagation makes Doppler effects significant even for relatively slowly moving platforms [3], resulting in decoding errors. In an effort to improve the performance of UWA communication, the received signal requires channel equalization processing and Doppler compensation.

TRM has temporal and spatial focusing capability. The temporal compression recombines the multipath signal and mitigates ISI, while the spatial focusing improves the signal-to-noise ratio (SNR) and reduce the influence of channel degradation. The capability of TRM makes it widely used in UWA communication [4,5,6,7,8,9]. However, there are still some residual ISI after TRM processing. Subsequently TRM combined with a single-channel decision feedback equalizer (DFE) was put forward to eliminate the residual ISI and achieves nearly optimal performance in theory [2,10,11,12]. The traditional TRM approach described above consists of a vertical receiving array, which complicates the communication device. In this paper, we use single element TRM, which consists of a single sound pressure sensor. Although the single element TRM sacrifices the spatial focusing gain of the array, it can still achieve temporal focusing and channel equalization by recombining the multipath channel structure [13,14,15]. During UWA communication, if there is relative motion between the transmitter and receiver, the communication signals will experience a Doppler compression or dilation [1], which will cause errors in decoding. Therefore, it is necessary to compensate Doppler. In [16,17,18], a phase-locked loop is used to estimate the Doppler coefficient. In [19,20], researchers estimate the Doppler coefficient by interleaving transmitting signal with preambles, such as training symbols or pseudo-noise (PN) sequences. References [21,22] estimated the Doppler coefficient by measuring the length variation of the signal, which is called a block Doppler estimation algorithm. After obtaining the Doppler coefficient, those approaches remove the Doppler effects by resampling the received signal. In this paper, we propose a real-time symbol-based Doppler compensation (SBDC) method for pattern time delay shift coding (PDS) communication scheme [15,23,24,25]. The proposed method does not need to calculate the Doppler coefficient and resample the received signal, moreover, it can compensate Doppler in real-time.

In view of significant multipath and Doppler effects in UWA channels, this paper proposes a new UWA communication method using single element TRM combined with SBDC (TRM-SBDC) based on the PDS communication scheme. The proposed method can not only suppress ISI resulting from multipath spreads, but also overcome the Doppler effects induced by relative motion between the transmitter and receiver. The results of the simulations with real sounding channels and the UWA communication experiments conducted in the Songhua River in Harbin (China) verify the feasibility and robustness of the proposed method.

## 2. Theory of TRM-SBDC UWA Communication

### 2.1. The Process Chain of the Communication System

**Figure 1:**
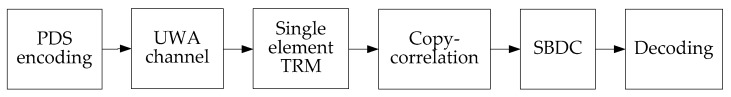
The flow chart of TRM-SBDC UWA communication system.

The paper applies the time reversal technique and SBDC technique to the PDS schemes and calls it the TRM-SBDC UWA communication system. Figure 1 shows the flow chart of the communication system. As shown in Figure 1, after the signal passes through the UWA channel, it is processed by TRM, so that the signal is temporally compressed and the ISI resulting from multipath spreads can be suppressed. Then, we use copy-correlation technique [15] to process the signal and get a series of correlation peaks, thereby calculating the time delay of each symbol. In addition, SBDC technique is performed to compensate the Doppler and help us obtain the correct decoding.

### 2.2. Principles of PDS

**Figure 2:**
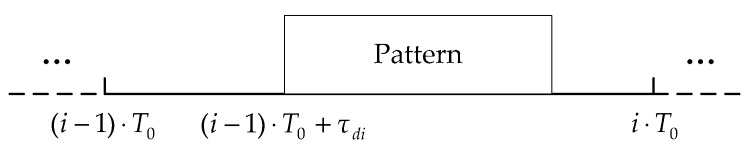
The symbol structure of PDS scheme.

The PDS scheme used in this paper belongs to the pulse position coding (PPC) scheme. Digital information is typically modulated in the code waveform, while the PDS scheme modulates the digital information in the time delay shift value of the pattern pulse. The different time delay shift values represent different information [23]. Figure 2 shows the symbol structure of the PDS scheme.

As shown in Figure 2, $\tau_{di}(i=1,2,3,\ldots )$ represents the time delay shift value, which is the position where the pattern pulse appears in the encoding window. $T_{0}$ is the length of one symbol, $T_{p}$ denotes the pattern pulse width, and $T_{c}=T_{0}-T_{p}$, which is the encoding time window for information coding. When each symbol carries n bits of information, the communication rate can be calculated as $n/T_{0}$. The quantization unit of time delay shift can be expressed as $\Delta \tau =T_{c}/\left(2^{n}-1\right)$, and the time delay shift value can be expressed as $\tau_{d}=k\cdot \Delta \tau$, where $k=0,1,\ldots ,2^{n}-1$. In this paper, each symbol carries 4 bits of digital information, so k=0 represents the digital information “0 0 0 0”, and k=10 represents the digital information “1 0 1 0”.

### 2.3. Principles of Single Element TRM

**Figure 3:**
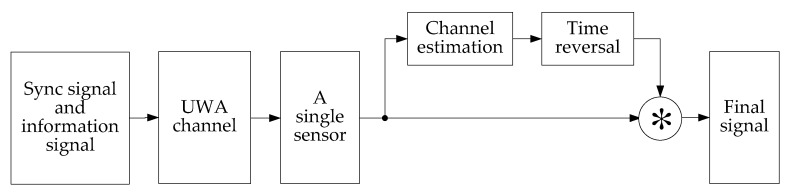
The block diagram of single element TRM.

The multipath spreads of UWA channels cause severe ISI, thereby degrading the quality of the communication signals, so it is necessary to perform channel equalization processing on the received signals. In this paper, the TRM based on a single sound pressure sensor is used to process the received signals. Although the single element TRM loses the spatial focusing gain of the vertical array, it can still undo the multipath or channel distortion and suppress ISI. Figure 3 illustrates the block diagram of single element TRM.

When performing time reversal communication, the transmitter usually sends a probe pulse signal to estimate the channel impulse response (CIR). The sync signal is used instead of the probe pulse signal in the paper, which is beneficial to save communication time and improve communication efficiency. The signal s(t) transmitted by the transmitter, including the sync signal p(t) and the information signal, passes through the UWA channel and is then received by the single sound pressure sensor. Assuming that the channel is stationary during the signal propagation, so the received signal can be expressed as:

(1) $$p_{r}(t)=p(t)*h(t)+n_{p}(t)\;$$

(2) $$s_{r}(t)=s(t)*h(t)+n_{s}(t)\;$$

where h(t) is CIR, $n_{p}(t)$ and $n_{s}(t)$ are underwater noises, and “∗” denotes convolution. Due to the multipath spreads, the CIR can be expressed as:

(3) $$h(t)=\sum_{i=1}^{N}A_{i}\delta (t-\tau_{i})\;$$

where N is the number of multipath between the transmitter and receiver, and $A_{i}$ and $\tau_{i}$ are the amplitude and time delay of the ith path.

The sync signal is intercepted from the received signal and processed by copy-correlation technique. Then the CIR estimation denoted as $h^{\prime }(t)$ is output. Finally, the received signal is convoluted with the time-reversed channel $h^{\prime }(-t)$ to obtain the final signal r(t), which can be expressed as:

(4) $$\begin{array}{ll} r(t) & =s_{r}(t)*h^{\prime }(-t)\\ & =s(t)*h(t)*h^{\prime }(-t)+n_{s}(t)*h^{\prime }(-t)\\ & =s(t)*\hat{h}(t)+n(t) \end{array}$$

where $\hat{h}(t)=h(t)*h^{\prime }(-t)$ denotes the virtual UWA channel through which the signal ultimately passes, and $n(t)=n_{s}(t)*h^{\prime }(-t)$ denotes the noise component. From a signal processing point of view, the virtual UWA channel is a cross-correlation function between the actual CIR and the estimated CIR. When the estimated CIR and the actual CIR match well, the energy of multipath signal is superimposed and the temporal focusing is achieved. In an ideal case, the virtual UWA channel approaches a delta function with a strong correlation peak and some low side lobes. That is to say, the single element TRM compresses the multipath structure, so that the virtual UWA channel can be viewed as a single path channel, thus the ISI is suppressed.

### 2.4. Principles of Copy-Correlation

Actually, copy-correlation can be viewed as a matched filter that can achieve a maximum SNR gain under ideal conditions. In the paper, we use copy-correlation technique to estimate the UWA acoustic channel and calculate the time delay shift value of each symbol.

According to Equations (1) and (3), the received sync signal can be expressed as:

(5) $$p_{r}(t)=\sum_{i=1}^{N}A_{i}p(t-\tau_{i})+n_{p}(t)\;$$

after copy-correlation processing, the output is:

(6) $$\begin{array}{ll} R(t) & =\int p_{r}(\tau )p(\tau -t)d\tau \\ & =\int \left[\sum_{i=1}^{N}A_{i}p(\tau -\tau_{i})\right]p(\tau -t)d\tau +\int n_{p}(\tau )p(\tau -t)d\tau \\ & =\sum_{i=1}^{N}A_{i}\int p(\tau -\tau_{i})p(\tau -t)d\tau +n_{p}^{\prime }(t) \end{array}$$

where $n_{p}^{\prime }(t)=\int n_{p}(\tau )p(\tau -t)d\tau$, denotes the noise component. We can see that copy-correlation outputs a series of correlation peaks, and the positions of the peaks corresponding to the time delay of the multiple paths. According to the correlation peaks and their positions, we can obtain the channel estimation.

Then the TRM is performed to compresses the multipath structure, and in an ideal case, the final sync signal can be expressed as:

(7) $$p_{r}^{\prime }(t)=\sum_{i=1}^{N}A_{i}p(t-\tau_{1})+n_{p}^{\prime \prime }(t)\;$$

and copy-correlation outputs only one correlation peak at $t=\tau_{1}$, which is the time delay of the sync code. Similarly, if the received signal is subjected to copy-correlation processing using the pattern pulse signal, we can obtain the correlation peaks of the symbols and calculate the time delay shifts.

### 2.5. Principles of SBDC

In the presence of relative motion between the transmitter and receiver in fluctuating underwater environments, the signals will be compressed or dilated due to Doppler effects. As a result, the time delay shift of the encoded pulse will be reduced or increased, that is, a time delay error will occur in each symbol. Moreover, the time delay error will be superimposed in PDS communication scheme, eventually leading to decoding errors. We propose a SBDC method to improve the performance of PDS communication in this paper.

**Figure 4:**
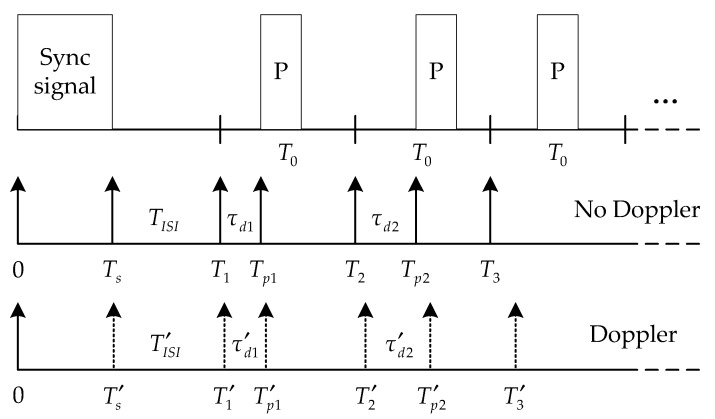
The diagram of decoding.

Since the received signal has been channel equalized by TRM, we use the copy-correlation technique for decoding [15,24]. First, the position of the sync signal can be found by correlating the received signal with the entire know sync signal, thereby the start position of the information code is determined. Subsequently, the received signal is subjected to copy-correlation processing using the pattern pulse signal and a series of correlation peaks are obtained. According to the positions of the correlation peaks, the time delay shift of each symbol can be calculated, thereby the information is demodulated. Figure 4 illustrates the schematic diagram of decoding.

As shown in Figure 4, $T_{s}$ is the pulse width of the sync signal, $T_{ISI}$ is the ISI guard time between the sync signal and the first symbol, $T_{i}$ denotes the start position of ith symbol, and $T_{pi}$ denotes the position of the pattern pulse. When there is no Doppler, $T_{i}$ can be determined by the position of the sync signal, and $T_{pi}$ can be obtained by the copy-correlation processing. So the time delay shift of the symbol is:

(8) $$\tau_{di}=T_{pi}-T_{i}=k_{i}\cdot \Delta \tau \;$$

where $k_{i}$ represents the digital information carried by the ith symbol, and $k_{i}=0,1,\ldots ,2^{n}-1$. However, $T_{i}$ and $T_{pi}$ will change if there is a Doppler effect, resulting in time delay errors. Assuming that the time delay error induced by the Doppler effect on the ith symbol is $\Delta_{Di}$, it can be defined as:

(9) $$\Delta_{Di}=\tau_{di}-\tau_{di}^{\prime }\;$$

In addition, it is considered that within the duration of each symbol the Doppler time delay error remains almost invariable, so the changed position of the pattern pulse can be expressed as:

(10) $$T_{pi}^{\prime }=T_{pi}-\sum_{j=1}^{i}\Delta_{Dj}-\Delta_{D0}\;$$

where $\Delta_{D0}$ is the time delay error caused by Doppler during the time period of the sync code. If the time delay shift is still calculated according to the start position of each symbol determined by the sync signal, the time delay shift of the ith symbol should be rewritten as:

(11) $$\begin{array}{ll} \tau_{di}^{\prime } & =T_{pi}^{\prime }-T_{i}\\ & =T_{pi}-\sum_{j=1}^{i}\Delta_{Di}-\Delta_{D0}-T_{i}\\ & =\tau_{di}-\sum_{j=1}^{i}\Delta_{Di}-\Delta_{D0} \end{array}$$

As can be seen from Equation (11), the time delay error will gradually increase. Once the time delay error accumulation exceeds Δτ/2, the decoding will generate errors. According to the decoding characteristics of the PDS communication scheme, the paper puts forward SBDC method. The proposed method uses the Doppler time delay error of the current symbol to compensate the time delay shift of the latter symbol, and has real-time performance.

The decoding process using SBDC is explained below. For the first symbol, it is assumed that the time delay error caused by Doppler is not enough to make the decoding error, so the time delay shift of the first symbol can be obtained as:

(12) $$\tau_{d1}^{\prime }=T_{p1}^{\prime }-T_{1}\;$$

According to Equation (9), the Doppler time delay error of the first symbol can be calculated as:

(13) $$\Delta_{D1}^{\prime }=\tau_{d1}-\tau_{d1}^{\prime }=\Delta_{D0}+\Delta_{D1}=k_{1}\cdot \Delta \tau -\tau_{d1}^{\prime }\;$$

then $\Delta_{D1}^{\prime }$ is used for Doppler compensation of the second symbol, we can obtain:

(14) $$T_{2}^{\prime }=T_{2}-\Delta_{D1}^{\prime }=T_{2}-\Delta_{D1}-\Delta_{D0}\;$$

(15) $$\tau_{d2}^{\prime }=T_{p2}^{\prime }-T_{2}^{\prime }=\tau_{d2}-\Delta_{D2}\;$$

(16) $$\Delta_{D2}^{\prime }=\Delta_{D2}=k_{2}\cdot \Delta \tau -\tau_{d2}^{\prime }\;$$

and so on, after the SBDC processing, the start position of the ith symbol is:

(17) $$\begin{array}{ll} T_{i}^{\prime } & =T_{i}-\sum_{k=1}^{i-1}\Delta_{Di}^{\prime }\\ & =T_{i}-\sum_{k=1}^{i-1}\Delta_{Di}-\Delta_{D0} \end{array},\;i\geq 2\;$$

thus the corresponding time delay shift and time delay error resulting from Doppler are respectively expressed as:

(18) $$\begin{array}{ll} \tau_{di}^{\prime } & =\tau_{di}-\Delta_{Di}\\ & =k_{i}\cdot \Delta \tau -\Delta_{Di} \end{array},\;i\geq 2\;$$

(19) $$\Delta_{Di}^{\prime }=\Delta_{Di},\;i\geq 2\;$$

Comparing Equations (11) and (18), each symbol obviously only needs to withstand its respective Doppler time delay error after SBDC, rather than the error accumulation of the preceding symbols. Consequently, no decoding error will occur as long as the Doppler time delay error of each symbol is within the error tolerance:

(20) $$-\Delta \tau /2<\Delta_{Di}^{\prime }<\Delta \tau /2\;$$

To analyze the performance of SBDC, we assume that the Doppler coefficient is α(t), and it is defined as:

(21) α(t)=v(t)/c(t) 

where v(t) is the relative radial speed between the transmitter and receiver, and c(t) is the sound speed in the water. Then the time delay error can be expressed as:

(22) $$\{\begin{array}{cc} \Delta_{D0}=\alpha (t)\cdot (T_{s}+T_{ISI}), & i=0\\ \Delta_{Di}=\alpha (t)\cdot T_{0}, & i\geq 1 \end{array}\;$$

For the PDS communication scheme, the key to correct decoding is to ensure that the time delay error of each symbol satisfies the Equation (20). The corresponding expression is:

(23) $$\{\begin{array}{cc} \left|\Delta_{D1}^{\prime }\right|=\left|\Delta_{D0}+\Delta_{D1}\right|<\Delta \tau /2, & i=1\\ \left|\Delta_{Di}^{\prime }\right|=\left|\Delta_{Di}\right|<\Delta \tau /2, & i\geq 2 \end{array}\;$$

According to Equations (21)–(23), we can get:

(24) $$\left|\alpha (t)\right|<\frac{\Delta \tau }{2(T_{s}+T_{ISI}+T_{0})}\;$$

(25) $$\left|v(t)\right|<\frac{\Delta \tau }{2(T_{s}+T_{ISI}+T_{0})}\cdot c(t)\;$$

that is to say, as long as the relative radial speed between the transmitter and receiver satisfies the Equation (25), the SBDC method will obtain a good decoding performance.

## 3. Simulations

Watermark [26] is a realistic simulation tool which is available for UWA communications. It is based on a replay channel simulator driven by measurements of the time-varying impulse response. KAU2 in Watermark is a single-input-multiple-output (SIMO) channel with a mean Doppler shift of about 1.28 m/s, and it contains 16 hydrophone channels. We use the hydrophone channel 4, 8 and 12 to validate the proposed method. Table 1 indicates the detailed parameters of an input packet. And the signal type is linear frequency modulation (LFM) signal which has good Doppler tolerance.

**Figure 5:**
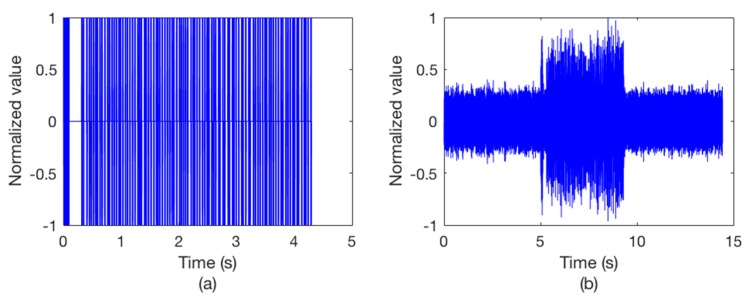
The waveforms of the packets. (**a**) The input packet; (**b**) the output packet after bandpass filtering, with a SNR of 5 dB in the frequency band.

We send the packet into Watermark and then it outputs 7 packets (2800 bits) for each hydrophone channel. The packet 1 of hydrophone channel 8 is taken example to describe the simulations. Figure 5 shows the clean input packet and the same packet after transmission through the hydrophone channel. The output signal is seriously distorted.

**Figure 6:**
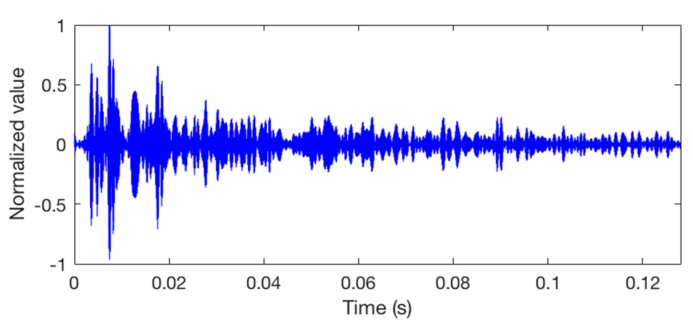
The channel impulse response of the packet.

**Figure 7:**
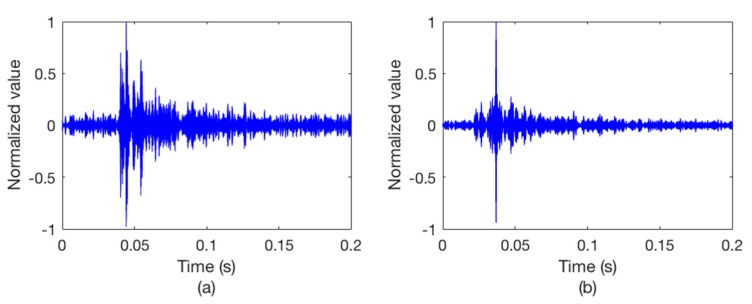
The outputs of the synchronous detection. (**a**) Without TRM; (**b**) with TRM.

After we get the Watermark output signal, copy-correlation technique is used to estimate the channel. Figure 6 shows the CIR at a certain moment. It can be seen that the hydrophone channel is characterized by a dispersive and multipath environment which causes the signal distortion. Therefore, we use a single element TRM to process the output packet. Figure 7 illustrates the outputs of the synchronous detection, wherein Figure 7a without using TRM while Figure 7b using TRM. Obviously, the energy of the sync signal is focused after the TRM processing and the position of the sync signal can be easily determined.

**Figure 8:**
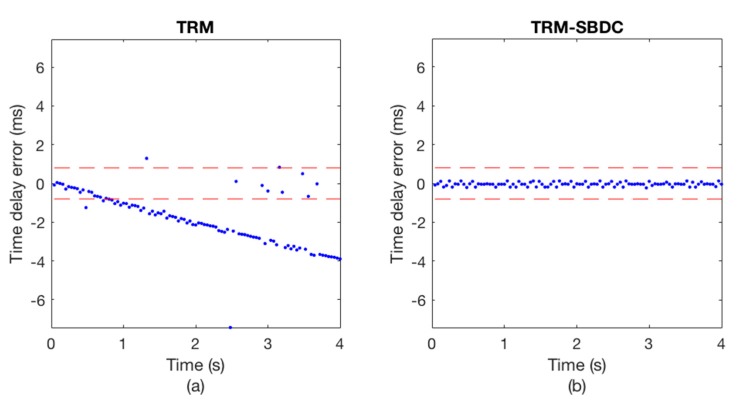
The Doppler time delay error. (**a**) TRM; (**b**) TRM-SBDC.

**Figure 9:**
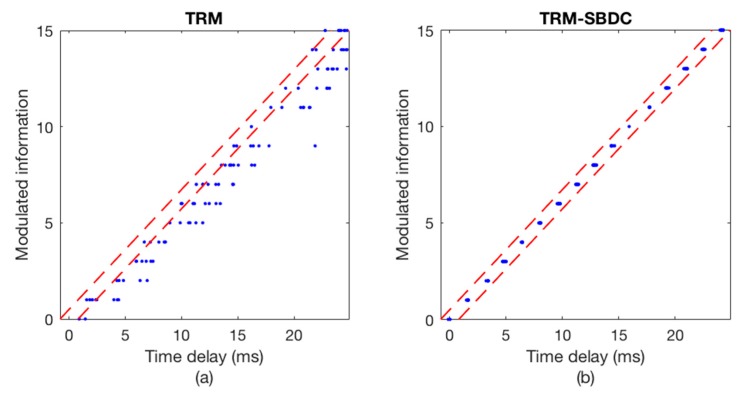
The decoding results. (**a**) TRM; (**b**) TRM-SBDC.

The communication results are illustrated in Figure 8 and Figure 9, wherein Figure 8 shows the Doppler time delay error and Figure 9 shows the decoding results. We can see that the time delay errors calculated without using SBDC are gradually increasing as shown in Figure 8a, leading to the decoding errors in Figure 9a. On the contrary, with SBDC processing, the time delay errors are all within the error tolerance range as shown in Figure 8b and no decoding error occurs as shown in Figure 9b.

Table 2 shows the communication performance of different hydrophone channels. As can be seen, the performance is greatly improved by the proposed method.

## 4. Experiments

In order to verify the feasibility and robustness of TRM-SBDC communication method proposed in the paper, an UWA communication experiment was conducted in the Songhua River in Harbin, in May 2018. The water depth in the experimental area was about 2 m, the sound velocity was about 1487 m/s, the maximum water flow rate was 1.8 m/s, and it was windy during the experiment. A single sound pressure sensor was deployed off the receiving boat which was always anchored, while the sending boat which has a sound source was moored or drifting with the current. Both the receiving sensor and the sound source were deployed to 1 m depth. During the experiment, we measured the speed using a handheld GPS device. In case of drifting with the current, the relative radial velocity between the two boats was about 1.0–1.8 knots. For the communication signal, the detailed parameters were the same as the signal in the simulations and the data length was 4000 bits. Table 3 shows the experimental scenes.

**Figure 10:**
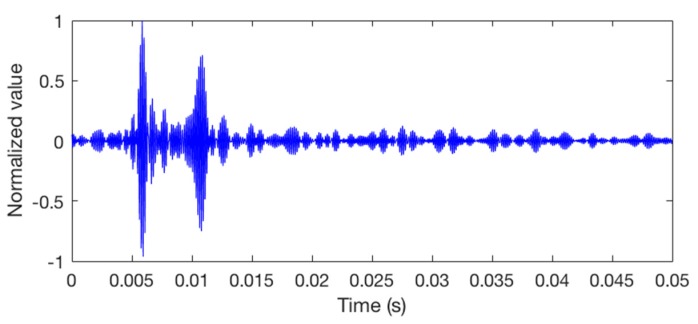
The CIR corresponding to a certain frame data.

A probe pulse signal is usually sent first to estimate the UWA channel. However, this experiment uses the sync signal instead of the probe pulse signal to estimate CIR, thereby improving the communication efficiency. Figure 10 illustrates the CIR corresponding to a certain frame data. It can be seen that the channel is characterized by serious multipath spreads, which is easy to cause ISI in communication signal.

**Figure 11:**
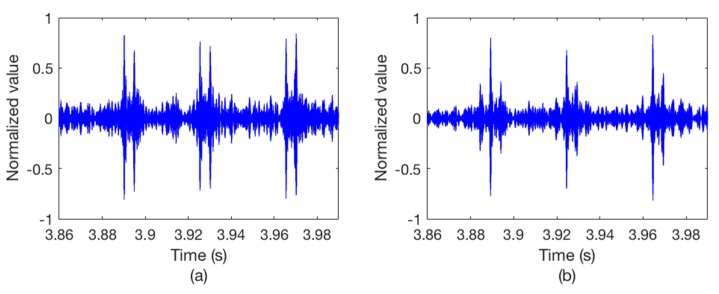
The copy-correlation results of the corresponding data. (**a**) Without TRM; (**b**) with TRM.

**Figure 12:**
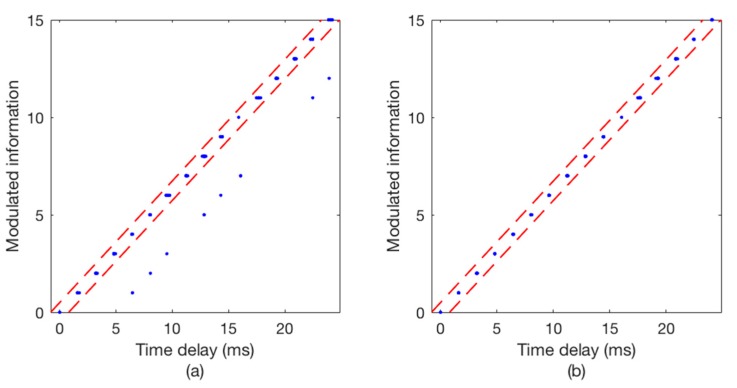
The decoding results of the corresponding data. (**a**) Without TRM; (**b**) with TRM.

Figure 11 illustrates the copy-correlation outputs of the corresponding communication data, wherein Figure 11a without using TRM and Figure 11b using TRM. For the convenience of observation, only partial waveforms of the copy-correlation outputs are displayed. Obviously, without TRM processing, there are strong pseudo correlation peaks with a delay of about 5 ms, which will confuse the judgment of the real correlation peaks and lead to decoding errors. On the contrary, the signal energy is focused after TRM processing, and the positions of the true correlation peaks are complete clear, thus ensuring the correctness of decoding. Figure 12 shows the corresponding decoding results, including Figure 12a without using TRM and Figure 12b using TRM. As can be seen from Figure 12a, the time delay errors of the error codes are all about 5 ms, which is consistent with the CIR in Figure 10 and the copy-correlation outputs in Figure 11a. However, the decoding results shown in Figure 12b are greatly improved, indicating that the single element TRM used in this paper is simple and effective.

**Figure 13:**
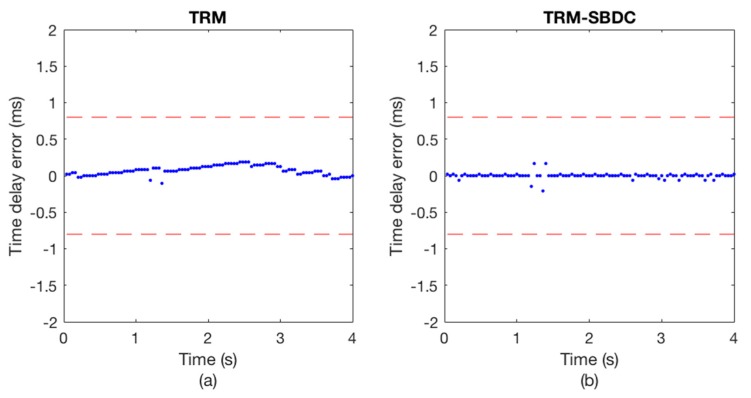
The Doppler time delay error when the sending boat is moored. (**a**) TRM; (**b**) TRM-SBDC.

**Figure 14:**
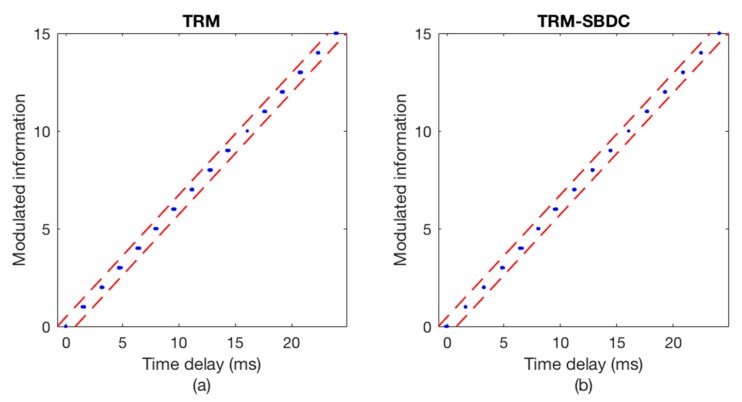
The decoding results when the sending boat is moored. (**a**) TRM; (**b**) TRM-SBDC.

In experimental scene 1, the sending boat is moored. Figure 13 and Figure 14 show the communication results of a certain frame data in this scene. Figure 13a depicts the Doppler time delay errors calculated using only TRM, while Figure 13b depicts the Doppler time delay errors calculated using TRM-SBDC. It can be seen that even if the sending boat and the receiving boat are anchored, the ups and downs of the boats caused by the wind and waves will still induce weak Doppler. However, since the time delay errors caused by Doppler are within the error tolerance range, no error occurs in the decoding results, as shown in Figure 14. Wherein Figure 14a shows the decoding results using only TRM, while Figure 14b shows the decoding result using TRM-SBDC.

**Figure 15:**
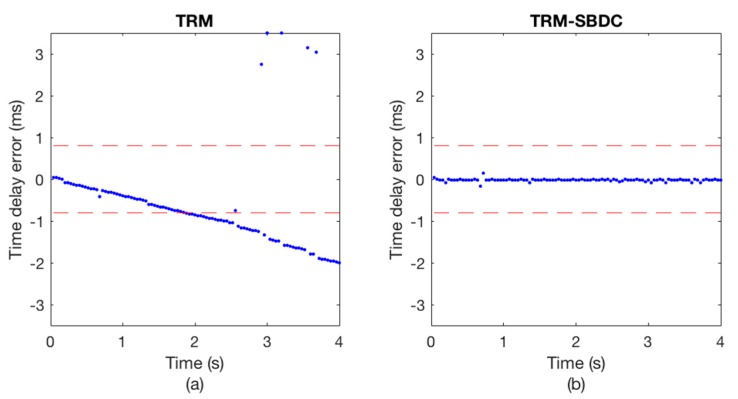
The Doppler time delay error when the sending boat is drifting. (**a**) TRM; (**b**) TRM-SBDC.

**Figure 16:**
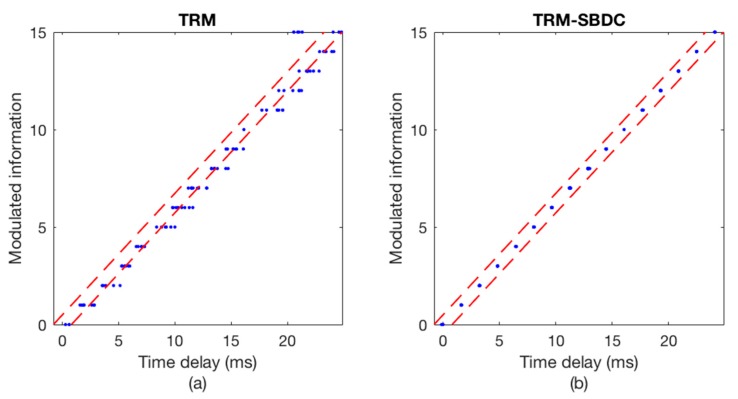
The decoding results when the sending boat is drifting. (**a**) TRM; (**b**) TRM-SBDC.

A certain frame data from experimental scene 4 is used to illustrate the communication performance as shown in Figure 15 and Figure 16. The sending boat is drifting with the current in experimental scene 4. Figure 10a shows the time delay errors calculated without using SBDC, while Figure 15b shows the time delay errors calculated using TRM-SBDC. Obviously, when the sending boat is drifting with the water flow, the relative motion between the sending boat and the receiving boat makes Doppler effects become significant. Moreover, without Doppler compensation, the Doppler time delay errors are gradually accumulated and finally exceed the error tolerance range as shown in Figure 15a. According to the Doppler time delay errors, the average relative radial velocity is about 1.5 knots. Figure 11 illustrates the corresponding decoding results, wherein Figure 16a shows the decoding results using only TRM, and there are many decoding errors, while Figure 16b shows the decoding results using TRM-SBDC, and no error occurs.

Table 4 shows the decoding results of UWA communication of each experimental scene. As can be seen, Doppler effects lead to a large number of decoding errors, but the performance of each experimental scene is greatly improved after the SBDC processing, indicating that the TRM-SBDC technique is practical and robust.

## 5. Conclusions

Aiming at the addressing the issues of multipath spreads and Doppler effects in underwater environments, this paper proposes the TRM-SBDC UWA communication method to improve the performance of UWA communications. TRM based on a single sound pressure sensor is used to reform the multipath structure and achieve energy focusing of the signal, so as to mitigate the ISI. Subsequently, the Doppler effect of each symbol is compensated in real-time by the SBDC method, thereby reducing the time delay error and obtaining a low BER. The results of simulations with real sounding channels indicate that the proposed method can greatly improve the UWA communication performance. In UWA communication experiments conducted in the Songhua River, Harbin, the proposed method achieved nearly error-free performance with a communication rate of 100 bit/s in the 5.5–7.5 kHz band, confirming the feasibility and robustness of the TRM-SBDC communication method, and indicating that the method proposed in this paper could be of great significance for mobile UWA communications.

## Figures and Tables

**Table 1:** Parameters of the communication signal.

Parameter	Value
*T_s_* (ms)	100
*T_ISI_* (ms)	200
*T_p_* (ms)	16
*T_c_* (ms)	24
Bandwidth (kHz)	2(5.5–7.5)
*n* (bit)	4
Information size (bit)	400
Communication rate (bit/s)	100

**Table 2:** The decoding results of different hydrophone channels.

Hydrophone Channels of KAU2	BER (%)	
	TRM	TRM-SBDC
Hydrophone channel 4	34.000	0
Hydrophone channel 8	30.893	0.321
Hydrophone channel 12	34.036	1.429

**Table 3:** Introduction to experimental scenes.

Experimental Scene	Initial Distance (m)	Status of the Sending Boat
1	450	moored
2	705	drifting
3	640	drifting
4	690	drifting

**Table 4:** The decoding results of different experimental scenes.

Experimental Scene	BER (%)	
	TRM	TRM-SBDC
1	0	0
2	15.325	0
3	26.550	0.050
4	24.025	0

## References

[1] Kilfoyle D.B., Baggeroer A.B. (2000). The state of the art in underwater acoustic telemetry. IEEE J. Ocean. Eng..

[2] Song H.C., Hodgkiss W.S., Kuperman W.A., Stevenson M., Akal T. (2006). Improvement of time-reversal communications using adaptive channel equalizers. IEEE J. Ocean. Eng..

[3] Song H.C. (2016). An overview of underwater time-reversal communication. IEEE J. Ocean. Eng..

[4] Edelmann G.F., Akal T., Hodgkiss W.S., Kim S., Kuperman W.A., Song H.C. (2002). An initial demonstration of underwater acoustic communication using time reversal. IEEE J. Ocean. Eng..

[5] Yang T.C. (2003). Temporal resolutions of time-reversal and passive-phase conjugation for underwater acoustic communications. IEEE J. Ocean. Eng..

[6] Song H.C. (2013). Time reversal communication with a mobile source. J. Acoust. Soc. Am..

[7] Shimura T., Watanabe Y., Ochi H., Song H.C. (2012). Long-range time reversal communication in deep water: Experimental results. J. Acoust. Soc. Am..

[8] Shimura T., Ochi H., Song H.C. (2013). Experimental demonstration of multiuser communication in deep water using time reversal. J. Acoust. Soc. Am..

[9] Song H.C., Howe B., Brown M., Andrew R. (2014). Diversity-based acoustic communication with a glider in deep water. J. Acoust. Soc. Am..

[10] Shimura T., Kida Y., Deguchi M. Basic research on MIMO underwater communication using adaptive time reversal. Proceedings of the 2017 IEEE Oceans.

[11] Song A., Badiey M., Song H.C., Hodgkiss W.S., Poter M.B. (2008). Impact of ocean variability on coherent underwater acoustic communications during the Kauai experiment. J. Acoust. Soc. Am..

[12] Song H.C., Hodgkiss W.S. (2015). Self-synchronization and spatial diversity of passive time reversal communication. J. Acoust. Soc. Am..

[13] Yin J.W., Du P.Y., Shen J.W., Guo L.X. Study on time reverse mirror in underwater acoustic communication. Proceedings of the Meetings on Acoustics.

[14] Han X., Yin J.W., Du P.Y., Zhang X. (2014). Experimental demonstration of underwater acoustic communication using bionic signals. Appl. Acoust..

[15] Zhao A.B., Zeng C.G., Hui J., Ma L., Bi X.J. (2017). Experimental Demonstration of Long-Range Underwater Acoustic Communication Using a Vertical Sensor Array. Sensors.

[16] Johnson M., Freitag L., Stojanovic M. Improved Doppler tracking and correction for underwater acoustic communications. Proceedings of the 1997 IEEE International Conference on Acoustics, Speech and Signal Processing.

[17] Tu K., Duman T.M., Stojanovic M., Proakis J.G. (2013). Multiple-resampling receiver design for OFDM over Doppler-distorted underwater acoustic channels. IEEE J. Ocean. Eng..

[18] Song H.C., Hodgkiss W.S., Kuperman W.A., Higley W.J., Raghukumar K., Akal T., Stevenson M. (2006). Spatial diversity in passive time reversal communications. J. Acoust. Soc. Am..

[19] Kim Y.H., Song I., Yoon S., Park S.R. (2001). An efficient frequency offset estimator for OFDM systems and its performance characteristics. IEEE Trans. Veh. Technol..

[20] Tufvesson F., Edfors O., Faulkner M. Time and frequency synchronization for OFDM using PN-sequence preambles. Proceedings of the IEEE VTS 50th Vehicular Technology Conference.

[21] Sharif B.S., Neasham J., Hinton O.R., Adams A.E. (2000). A computationally efficient Doppler compensation system for underwater acoustic communications. IEEE J. Ocean. Eng..

[22] Luan Y.F., Yan S.F., Qin Y., Xu L.J. Doppler Estimation Using Time Reversal Mirror for Underwater Acoustic Time-varying Multipath Channel. Proceedings of the 2017 IEEE International Conference on Signal Processing, Communications and Computing (ICSPCC).

[23] Hui J.Y., Liu L., Liu H., Feng H.H. (1999). A study on pattern time delay coding underwater acoustic communication. Acta Acust..

[24] Yin J.W., Hui J.Y., Hui J., Yao Z.X., Wang Y.L. (2006). Underwater acoustic communication based on pattern time delay shift coding scheme. Ocean. Eng..

[25] Yin J.W., Zhang X., Zhu G.P., Tang S.Y., Sun H. (2016). Parametric array differential pattern time delay shift coding underwater acoustic communication in the under-ice environment. Chin. J. Acoust..

[26] van Walree P.A., Socheleau F.-X., Otnes R., Jenserud T. (2017). The watermark benchmark for underwater acoustic modulation schemes. IEEE J. Ocean. Eng..

